# CircPVT1 up‐regulation attenuates steroid‐induced osteonecrosis of the femoral head through regulating miR‐21‐5p‐mediated Smad7/TGFβ signalling pathway

**DOI:** 10.1111/jcmm.16294

**Published:** 2021-03-18

**Authors:** Yangquan Hao, Chao Lu, Baogang Zhang, Zhaochen Xu, Hao Guo, Gaokui Zhang

**Affiliations:** ^1^ Department of Osteonecrosis and Joint Reconstruction Honghui Hospital Xi’an Jiaotong University Health Science Center Xi’an China; ^2^ Shaanxi University of Chinese Medicine Xi’an China

**Keywords:** ceRNA, circPVT1, miR‐21‐5p, osteonecrosis, steroid

## Abstract

Steroid‐induced osteonecrosis of the femoral head (SIONFH) has been a common disease following corticosteroid therapy. Presently, we aim to explore the functions of circular RNA (circ) PVT1 in SIONFH rats and the underlying mechanism. Glucocorticoid (GC) was used to treat SD rats and bone marrow‐derived mesenchymal stem cells (BMSCs) to construct SIONFH model in vitro and in vivo, respectively. The pathological injury of the femoral head in the SIONFH rats was detected via haematoxylin‐eosin (HE) staining and immunohistochemistry (IHC). The osteogenic differentiation, proliferation and apoptosis of BMSCs were detected. Western blot was used to detect Smad7, Bax, Bcl2 and Smad2/3. The potential targets of circPVT1 and miR‐21‐5p were validated through luciferase reporter gene assay and RNA pull‐down assay, respectively. We found that CircPVT1 was decreased in the femoral head of SIONFH rats and GC‐treated BMSCs, while miR‐21‐5p was markedly up‐regulated. Overexpressed circPVT1 attenuated the apoptosis and cell viability inhibition of BMSCs induced by GC, while miR‐21‐5p up‐regulation had the opposite effects. What's more, the in vivo experiments confirmed that up‐regulating circPVT1 repressed osteonecrosis in SIONFH rats through repressing apoptosis. Mechanistically, circPVT1 functioned as a ceRNA of miR‐21‐5p, which targeted at the 3'untranslated region of Smad7. CircPVT1 enhancing Smad7 and mitigating GC activated TGFβ/Smad2/3 pathway through inhibiting miR‐21‐5p. In conclusion, CircPVT1 exerts protective effects against SIONFH via modulating miR‐21‐5p‐mediated Smad7/TGFβ pathway.

## 1 INTRODUCTION

Osteonecrosis of the femoral head (ONFH), as a progressive degenerative disease at the hip, is characterized as a microfracture of the subchondral bone and subsequent collapse of the femoral head, leading to hip dysfunction.^1^, ^2^ During the ONFH progression, many factors are involved in that process, including genetic factors and trauma, long‐term alcohol abuse, extensive use of steroids, long‐term diving operations and other environmental factors.^3^, ^4^ Among them, ONFH caused by excessive steroid use is the leading cause of non‐traumatic ONFH. Excessive use of steroids can lead to lipid metabolism disorder, vascular endothelial injury, medullary fat accumulation, lipid/osteoblastic disorderly differentiation of BMSCs, hindering bone repair and reconstruction.^5^, ^6^ However, because of insufficient early diagnosis, hip arthroplasty (BHA) is mostly performed to solve the pain and claudication problems in the middle and late stages. This situation imposes a heavy financial burden on society and families.^7^ Hence, with development of people's life, the early diagnosis and treatment of steroid‐induced osteonecrosis of the femoral head (SIONFH) have become an urgent target.

Circular RNA (circRNA), a long non‐coding endogenous circular RNA molecule without protein‐coding function, participates in various genome transcriptions and post‐transcriptional levels.^8^ In the late days, an increasing number of papers have reported that CircRNA affects gene expression, thus modulating the progression of orthopaedic diseases. Taking CircRNA‐9119 as an example, its overexpression up‐regulates PTEN expression by down‐regulating microRNA‐26a to protect IL‐1β‐treated osteoarthritis‐mediated chondrocytes from apoptosis.^9^ Besides, CircGCN1L1 promotes synovium cell proliferation and chondrocyte apoptosis by targeting miR‐330‐3s and TNF‐α in TMJ osteoarthritis.^10^ CircPVT1 is an essential member of the circRNA family and has shown its important role in several tumours. For instance, the down‐regulation of CircPVT1 represses the growth of liver cancer cell via modulating miR‐3666/SIRT7 axis.^11^ Additionally, circPVT1 functions as a ceRNA of miR‐526b, thus promoting the metastasis of osteosarcoma via up‐regulation FOXC2.^12^ Kun‐Peng Z et al also found that circPVT1 enhances the chemoresistance of osteosarcoma cells to adriamycin and cisplatin through ABCB1.^13^ However, the distinct expression of CircPVT1 within SIONFH and its specific regulatory mechanism are still unknown.

MiRNAs, a class of endogenous single‐stranded non‐coding small RNAs, are 20‐24 nucleotides in length. Researchers have found that miRNAs modulate cell development and differentiation and play an increasingly significant role in protecting osteoblasts from invasion. Gu C et al demonstrated that up‐regulation of miR‐27a attenuated steroid‐induced adipose formation and promoted osteogenesis in rat's BMSCs by directly targeting PPARγ and GREM1.^14^ Similarly, Kong L et al reported in 2020 that silencing miR‐137‐3p can directly target Runx2 and CXCL12 and prevent SIONFH by promoting osteoblast differentiation and angiogenesis.^15^ MiR‐21‐5p, as an important member of the microRNA family, is located at 17q21.1 with 72 bp in length. Also, papers have shown that miR‐21‐5p has a significant effect on improving cognitive impairment,^16^ regulating extracellular matrix degradation and angiogenesis,^17^ but its role in SIONFH has not been reported.

Transforming growth factor β (TGFβ) is a multifunctional cytokine, including three receptors TGFβ R‐I, TGFβ R‐II and TGFβ R‐III. Among them, only type I and type II are necessary for the Smad signalling pathway and have membrane‐bound serine and threonine activity.^18^ Smad protein is the only known intracellular kinase substrate of TGFβ receptor, and it is a signal transduction factor downstream of TGFβ receptor.^19^ Smad7, one of Smad's proteins, acts as an intracellular inhibitory protein that antagonizes signal transduction between the TGF‐β family. Researches prove that Huogu I formula (I) can modulate the expression of BMP2, TGFβ/Smads and OPG/RANKL, thus promoting the repair of necrotic bones.^20^ Additionally, the study conducted by Bai et al suggested that miR‐27a regulates steroid‐induced ONFH via TGF‐β/Smad7 signalling.^21^ Collectively, the above studies suggest that TGF‐β/Smad7 signalling pathway plays an important role in SIONFH.

Here, both in vivo and in vitro SIONFH models were established to explore the specific role and mechanism of circPVT1 regulating SIONFH. We found circPVT1 was down‐regulated in the femoral head of rats with osteonecrosis as well as GC‐treated BMSCs. Up‐regulating circPVT1 significantly reduced GC‐mediated apoptosis of BMSCs. Additionally, the bioinformatic analysis showed that miR‐21‐5p is a potentially target at the downstream of circPVT1 and miR‐21‐5p was inhibited following circPVT1 up‐regulation. Therefore, we supposed that the circPVT1/miR‐21‐5p axis might exert a vital role in SIONFH via modulating the Smad7/TGFβ pathway. We hope that this finding provides a new reference for the prognosis and molecular targeted therapy of SIONFH.

## 2 METHODS AND MATERIALS

### 2.1 Establishment of SIONFH model

The experimental animal centre of Honghui Hospital Xian Jiao Tong University Health Science Center provided 10‐week‐old male SD rats. The Animal Ethics Committee of Honghui Hospital Xian Jiao Tong University Health Science Center approved all experimental procedures in this study. In twelve hours light and dark circle, all rats were free to drink and eat. We randomly divided them into a SIONFH model group (N = 5) and a control group (N = 5). The SIONFH model was described as^22^ and implemented after modification. Rats in the SIONFH model group were injected subcutaneously at day 1 with imiquimod (IMI 30 mg/kg), and intramuscularly at day 2 with methylprednisolone (MPSL 20 mg/kg). At the fourth week, all injections were made again. At the same time‐point, the model group and the control group were injected with the same amount of normal saline. Six weeks after the first injection, the rats were killed, and the femurs were collected.

### 2.2 BMSC isolation and culture

During total hip replacement, bone marrow tissue (5‐10 mL) was removed from the proximal femur with a sterile syringe. The collected bone marrow tissue was transferred to a centrifuge tube containing phosphate‐buffered saline (PBS) solution and carefully removed with a pipette to form a cell suspension. Then, suspension was transferred to a centrifuge tube containing the same amount of leucocyte separation liquid for centrifugation (30 minutes, 340 *g*). Next, the sample was resuspended in Dulbecco's Modified Eagle's Medium (DMEM; Gibco, Rockville, MD, USA) containing 10% foetal bovine serum (FBS; Gibco, Rockville, MD, USA). The cells were evenly inoculated in 6‐well plates and grew in an incubator (37°C, 5% CO_2_, 95% humidity). The medium was changed every 3 days.

### 2.3 Cell transfection

Cells at the logarithmic growth stage were taken and planted in 6‐well plates (5 × 10^6^/well) after trypsinization and passage. The cells were transfected after stable growth. Using GenePharma (Shanghai, China), the CircPVT1 overexpressed plasmid (CircPVT1), miR‐21‐5p mimics were obtained. Then, the cells were incubated (37°C, 5% CO_2_) and transfected with Lipofectamine^®^ 3000 (Invitrogen; Thermo Fisher Scientific, Inc) reagent following the instructions of FuGENE^®^ HD Transfection Reagent (Roche, Shanghai, China). Twenty‐four hours after transfection, we extracted total cellular RNA for real‐time fluorescence quantitative PCR to monitor the expression changes of CircPVT1, miR‐21‐5p in cells after transfection.

### 2.4 qRT‐PCR

First, we used the TRIzol reagent to extract total RNA. Second, the RNA was reverse transcribed into cDNA using PrimeScript™ RT Reagent kit (Invitrogen, Shanghai, China) in line with the manufacturer's instructions. Bio‐Rad CFX96 quantitative PCR system and SYBR were utilized to perform qRT‐PCR in accordance with supplier's regulations. The conditions of qRT‐PCR were as follows: pre‐denatured at 95°C for 5 minutes, then denatured at 95°C for 15 seconds and annealed at 60°C for 30 seconds. GAPDH was the endogenous control for CircPVT1, ALP, RUNX2, CoL1a1, OCN, ACP5 and CTSK, and U6 was the endogenous control for miR‐21‐5p. The 2^(−ΔΔCt)^ method was applied for statistics. All qRT‐PCRs were repeated three times. Guangzhou Ruibo Company designed and synthesized the primers. Primer sequences of each gene were shown in Table 1.

**TABLE 1:** Primer sequences of each gene

Genes	Primer sequences (5′→3′)
miR‐21‐5p	forward:ACCACCGTAGCTTATCAGACTGA
	reverse:CAGTGCAGGGTCCGAGGT
CircPVT1	forward:ACATTTCCTGCTGCCGTTTT
	reverse:CTTGGAGGCTGAGGAGTTCA
ALP	forward:TGAACCGCAGGATGTGAACT
	reverse:GAAGAAGGGGTGCTACGTCC
RUNX2	forward:TGATTTAGGGCGCATTCCTCA
	reverse:AGGATTGTGTCTGCCTGGGA
Col1a1	forward:GGAGAGAGCATGACCGATGG
	reverse:GCTACGCTGTTCTTGCAGTG
OCN	forward:ATTGTGACGAGCTAGCGGAC
	reverse:TCGAGTCCTGGAGAGTAGCC
ACP5	forward:TCCCCAGCCCTTATTACCGT
	reverse:GGCTGACAAAGTCGTCGGAA
CTSK	forward:CCGTGGTGAGCTTTGCTCTA
	reverse:TGTTGTACTGCTTCCCGTGG
U6	forward:CTCGCTTCGGCAGCACA
	reverse:AACGCTTCACGAATTTGCGT
GAPDH	forward:TGATCTTCATGGTCGACGGT
	reverse:CCACGAGACCACCACCTACAACT

### 2.5 Western blot

Bone marrow‐derived mesenchymal stem cells (BMSCs)s were collected and washed 3 times with PBS, with the addition of 100‐200 μL RIPA lysates (Beyotime Biotechnology, Shanghai, China), and cells were lysed in ice water by ultrasound. And the Bradford method was utilized to determine protein concentration. Equal amounts of protein from each group were subjected to 10% SDS‐PAGE electrophoresis, and the protein on the gel was transferred to PVDF membranes (Millipore, Bedford, MA, USA). For one hour, the membranes were sealed at 4°C, then they were incubated with the addition of Anti‐Smad7 antibody(ab216428), Anti‐p‐Smad2 antibody (ab18834), Anti‐Smad2 antibody (ab40855), Anti‐p‐Smad3 antibody (ab52903), Anti‐Smad3 antibody (ab40854), Anti‐Bcl2 antibody (ab182858) and Anti‐Bax antibody (ab32503) (overnight, 4°C). Next, TBST was used to wash the membranes twice. They were incubated with fluorescein‐labelled goat anti‐rabbit (ab205718, 1:2500) at room temperature for 1 hour. Abcam (Cambridge, UK) supplied the above antibodies. After being washed three times, they were exposed with ECL developer (Millipore, Bedford, MA, USA) and imaged with a membrane scanner.

### 2.6 BrdU method for the cell viability

Single‐cell suspensions were made from BMSCs at the logarithmic growth stage and seeded on 24‐well plates (1 × 10^5^/well). After cells were attached to the walls, GC (1600 mg), miR‐21‐5p mimic and CircPVT1 were added, respectively, to each group. BrdU‐labelled reagent (AmyJet Scientific Inc, China) was added as per operation instructions and cultured in 5% CO_2_ and 37°C incubators. After 48‐hour continuous cell culture, the cells were stained with immunofluorescence. Under a microscope, the numbers of positive cells and DAPI‐positive cells in 3 fields were randomly selected and calculated. We took the average cell proliferation rate of 3 visual fields as the cell proliferation rate. Cell proliferation rate = the number of BrdU‐positive cells/number of DAPI‐positive cells.

### 2.7 Flow cytometry for cell apoptosis

Annexin V‐FITC double staining method was carried out to detect cell apoptosis. Twenty‐four hours after transfection, the BMSCs underwent trypsinization and collection and were inoculated in a 6‐well plate (2 × 10^6^ cells/well), followed with 24‐hour culture. When the supernatant was discarded, the cells were washed twice with pre‐cool PBS and resuspended using 1 × Binding buffer. Afterwards, with the addition of 5 μL Annexin V‐FITC and 5 μL PI, the cell suspension was thoroughly mixed and incubated (room temperature, 15 minutes). Within 1 hour, we carried out the flow cytometry to measure cell apoptosis rate. The cell apoptosis rate = number of apoptotic cells/(number of apoptotic cells + number of normal cells) × 100%. The steps were performed in line with the instructions of the apoptosis kit (purchased from Yeasen Biotech Co., Ltd., Shanghai, China).

### 2.8 HE staining

Bone tissue samples were secured with 10% neutral buffer formalin for 24 hours, decalcified for 4 weeks with 5% ethylenediaminetetraacetic acid. The solution was changed every 3 days. Samples of the SIONFH group and the control group were dehydrated with gradient ethanol (70%, 80%, 90%, 95% and 100%), permeated with xylene twice (5 minutes/time) paraffin‐embedded and sliced 4 μm. Then, the samples underwent the following operations: 1 hours bake at 80°C, dehydration with gradient ethanol, xylene permeation, haematoxylin staining (4 minutes), differentiation with hydrochloric alcohol (10 seconds), turn blue with ammonium hydroxide (10 minutes) and eosin staining (4 min). Then, the samples were dehydrated with gradient ethanol (1 minute/time), permeated twice with xylene (1 minute/time). Finally, the sections were sealed with neutral resin. We observed the morphological differences of the FHs cells in the SIONFH group and the control group using an optical microscope. As for HE staining in animal experiments, the above steps were duplicated. SIONFH histopathological changes were monitored under an optical microscope, and bone analysis parameters were measured by image analysis. We select three view fields from each section and calculated the average value.

### 2.9 TRAP staining

Tartrate‐resistant acid phosphatase (TRAP) staining was conducted to assess osteoclast activity using Acid Phosphatase, Leukocyte (TRAP) Kit form Sigma‐Aldrich. Methyl green was used to replace haematoxylin for cell nuclei staining, to give prominence to TRAP‐positive cells labelled in purplish red.

### 2.10 Immunohistochemical (IHC)

The specimens were fixed with 4% neutral formaldehyde solution, dehydrated, transparent, waxed, embedded, cut into 4 μ m thick sections, dehydrated with xylene and gradient ethanol, stained with haematoxylin for 5 minutes, 1‐3 seconds with 1% hydrochloric acid ethanol, 1‐3 minutes with 0.5% eosin solution, sealed with neutral gum and observed under light microscope. Leica (Bond max) automatic immunohistochemical staining machine was used for immunohistochemical staining. Anti‐Smad7 antibody (ab216428), anti‐Bax antibody (ab32503) and Abcam (Cambridge, UK) supplied the above antibodies were used to repair the tissue according to the instructions.

### 2.11 Dual‐luciferase reporter gene assay

The luciferase reporter gene assay was achieved using a dual‐luciferase reporter assay system (Promega, Madison, WI, USA). To construct pGL3‐CircPVT1‐wild‐type (CircPVT1‐WT) and pGL3‐CircPVT1‐mutant (CircPVT1‐MUT) reporter vector, the target fragments of wild‐type CircPVT1 and mutant CircPVT1 were constructed and integrated into pGL3 vector (Promega, Madison, WI, USA). Similarly, the target fragments of wild‐type Smad7 and mutant Smad7 were constructed and integrated into pGL3 vector (Promega, Madison, WI, USA) to construct pGL3‐Smad7‐wild‐type (Smad7‐WT) and pGL3‐Smad7‐mutant (Smad7‐MUT) reporter vector. BMSC cells were co‐transfected with CircPVT1/Smad7‐WT or CircPVT1/Smad7‐MUT and miR‐21‐5p or negative control. Following the manufacturer's instructions, luciferase activity was measured 48 hours after transfection. All experiments were made in triplicate and repeated three times.

### 2.12 RNA pull‐down assay

RNA pull‐down assay was studied by Pierce Magnetic RNA–Protein Pull‐Down Kit (Thermo Fisher Scientific, Waltham, MA, USA). Firstly, Bio‐miR‐21‐5p‐WT/Mut containing wild‐type or mutated seed regions, as well as their control Bio‐NC, were obtained by using the Biotin RNA Labeling Mix (Roche, Mannheim, Germany) and T7 RNA polymerase (Roche). Afterwards, cell lysates collected using RIPA lysis buffer were subjected to above biotin‐labelled miRNAs overnight. Then, we added magnetic beads coupled with streptavidin to pull down biotin‐labelled miRNAs and their interacting compounds. Eventually, RNAs (CircPVT1 or Smad7 mRNA) in each group's compounds were assayed using qRT‐PCR.

### 2.13 Data analysis

All statistical analyses were performed with SPSS22.0 statistical software (SPSS Inc, Chicago, IL, USA). The evaluation of the data was shown as mean ± standard deviation (x ± s). Univariate ANOVA was employed for the multi‐factor comparison, and independent sample *t* test was used for the comparison between two groups. All differences in performance were statistically significant when *P* < .05.

## 3 RESULTS

### 3.1 Bone histomorphology of SIONFH rats

**FIGURE 1:**
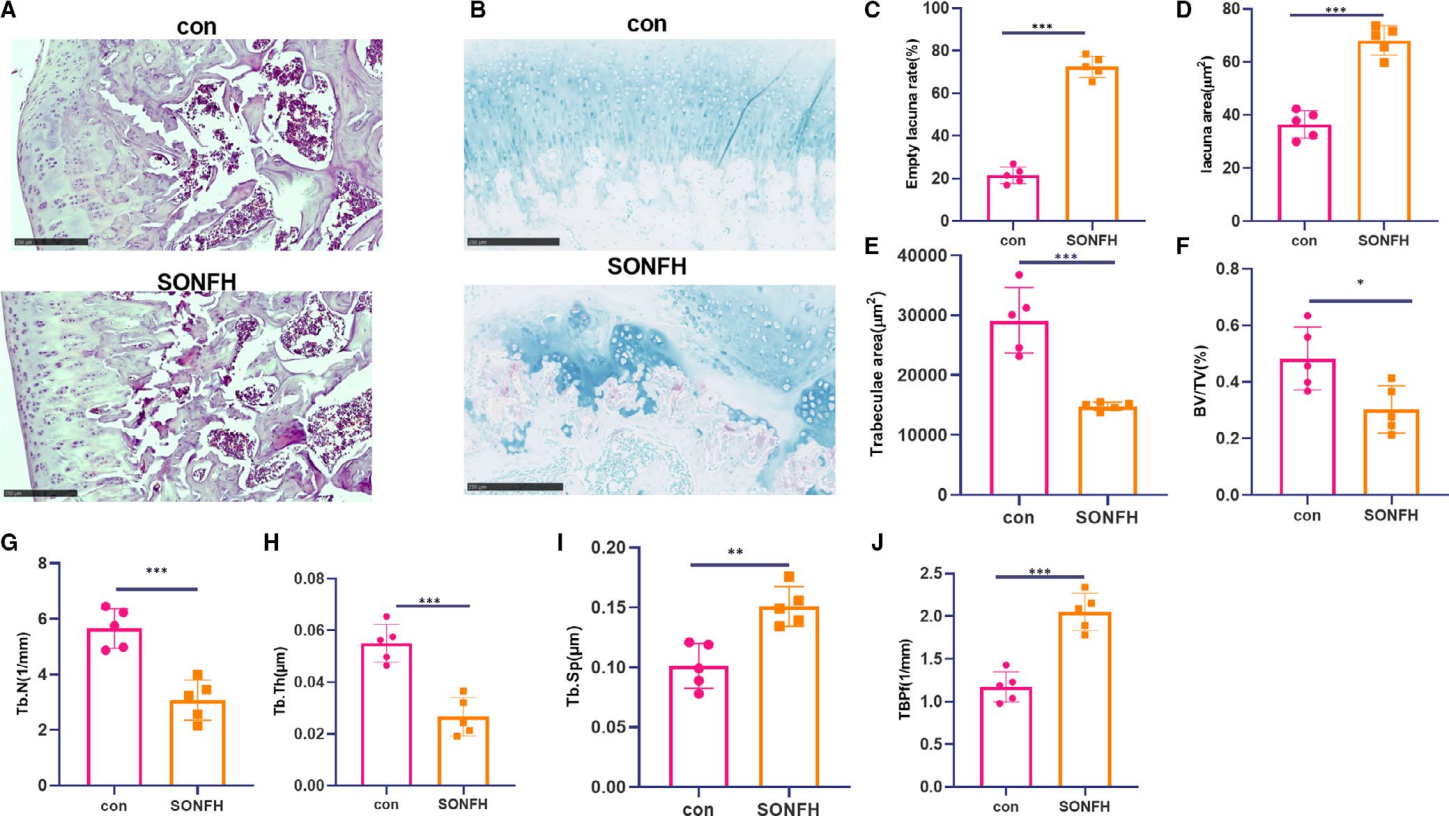
Bone histomorphology examination of SIONFH rats. The SIONFH model was constructed in rats. A, HE staining was taken to check the pathological damage of femur. B, TRAP staining was adopted to detect osteoclast viability. C‐E, Measurement of bone cavity rate, cavity area and trabecular area. F‐J, Determination of bone volume fraction, trabecular bone quantity, trabecular bone thickness, trabecular bone separation and mode factor. *P < .05, **P < .01, ***P < .001 (vs.con group). N = 5

Hormone mediation can have a severe effect on bone morphology, which may eventually lead to ONFH. We first examined the pathological injury of the bone by HE staining. It was found that the trabeculae in FH sections of normal rats were clearly visible and neatly arranged. Bone cells can be seen in the cortical bone and trabecular bone, and the cell nucleus was large. The bone lacuna was filled with bone cells. The calcification zone was well connected with the subchondral trabecular meshwork. However, the trabecular meshwork of the SIONFH rats became thinner or even broken. Fibrosis and granulation tissue hyperplasia can be seen around, and there were necrotic bone cells in the bone pockets, and there were no bone cells in a large number of cavities (Figure 1A) and the TRAP‐labelled osteoclasts were enhanced (Figure 1B). The measurement results of bone cavity rate, cavity area and trabecular area showed that compared with the standard group, the cavity rate and cavity area of the SIONFH group increased, and the trabecular area decreased (*P* < .05, Figure 1C‐E). At the same time, the bone volume fraction, trabecular bone number and thickness were notably reduced, while the trabecular bone separation and mode factor were significantly increased (*P* < .05, Figure 1F‐J). The results of bone histomorphology revealed that the femoral head's hormone‐induced necrosis severely damaged the typical morphology of bone tissue.

### 3.2 Effects of GC on osteogenic differentiation ability of BMSCs and expression level of CircPVT1/miR‐21‐5p

**FIGURE 2:**
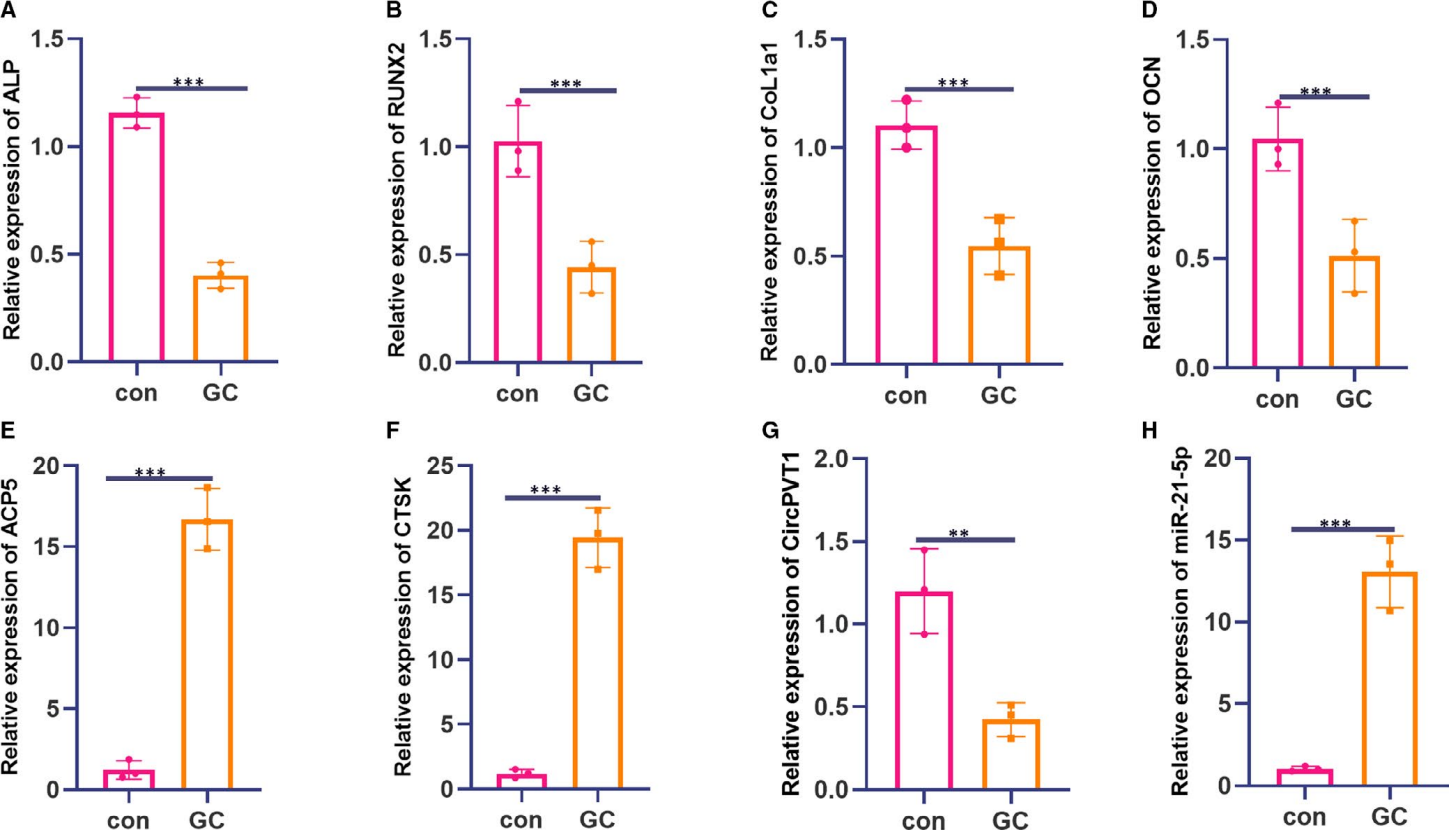
The effect of GC on the osteogenic differentiation ability of BMSCs and the expression level of CircPVT1/miR‐21‐5p. The BMSC was treated with 1600 mg of GC and tested their osteogenic differentiation ability. A‐D, RT‐PCR experiment was used to detect the levels of osteogenic differentiation‐specific markers ALP, RUNX2, CoL1a1 and OCN. E‐F, RT‐PCR experiment was applied to examine the expression of osteoclast‐related genes ACP5 and CTSK. G‐H, RT‐PCR was used to detect the expression of CircPVT1 and miR‐21‐5p in BMSCs. **P < .01, ***P < .001 (vs.con group). N = 3

First, we tested osteogenic differentiation markers and genes related to osteoclast formation. The RT‐PCR result illustrated that after 1600 mg GC treatment, the levels of osteogenic differentiation‐specific markers ALP, RUNX2, CoL1a1 and OCN were down‐regulated (compared with the con group) (*P* < .05, Figure 2A‐D). In comparison, the expression of osteoclast‐related genes ACP5 and CTSK were up‐regulated (*P* < .05, Figure 2E,F). Besides, the expression of CircPVT1 and miR‐21‐5p in BMSCs were examined via RT‐PCR experiment. It was found that after GC treatment, CircPVT1 expression was decreased, and miR‐21‐5p expression was increased (*P* < .05, Figure 2G,H). The above results indicated that the osteogenic differentiation ability of BMSCs was considerably suppressed by GC, and the osteoclast ability was significantly enhanced.

### 3.3 CircPVT1 overexpression promoted the differentiation and proliferation of BMSCs and inhibited cell apoptosis

**FIGURE 3:**
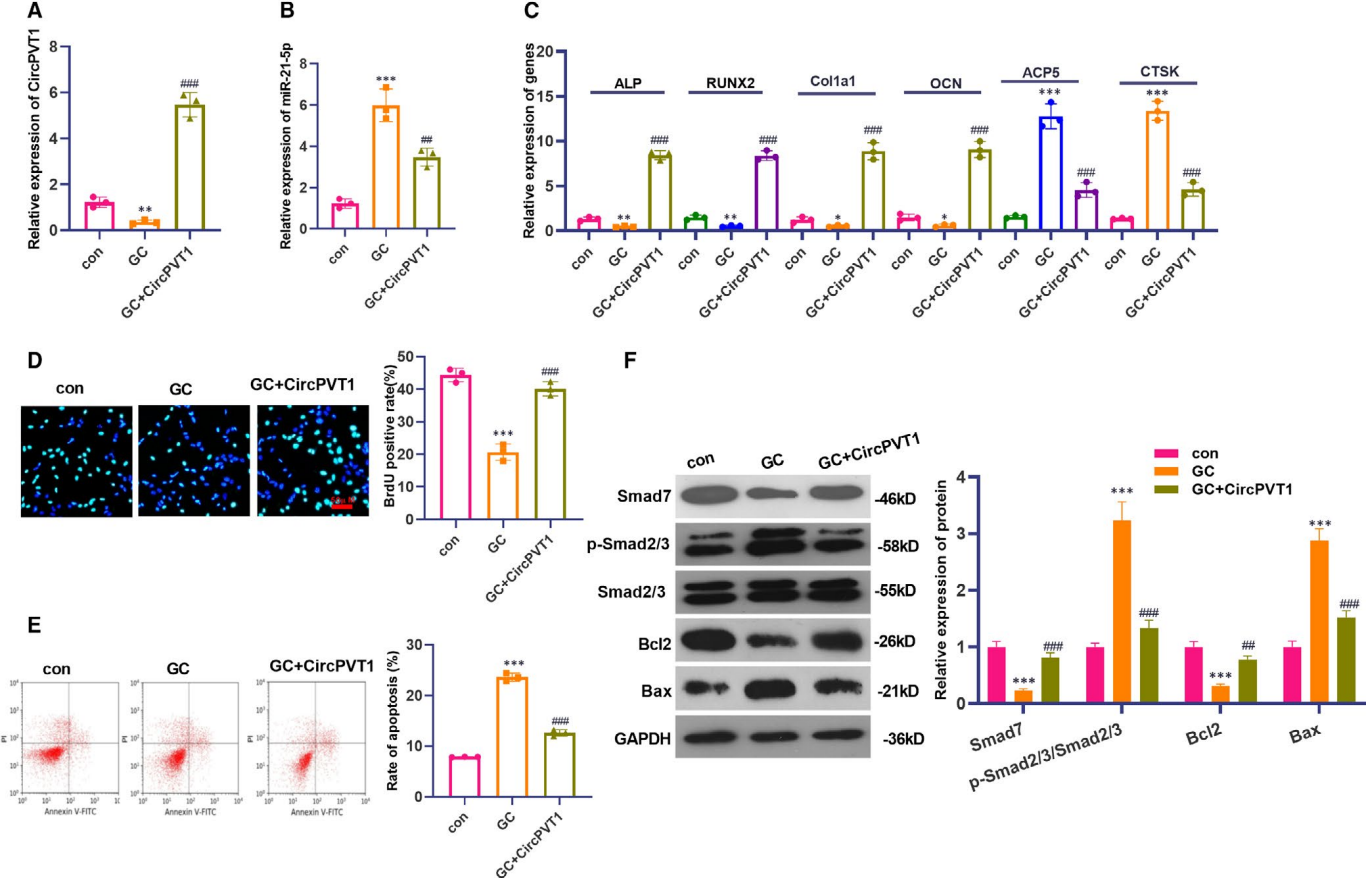
Overexpression of CircPVT1 promoted differentiation, proliferation and inhibited apoptosis of BMSCs. After 1600 mg of GC, CircPVT1 overexpression plasmid was also given to treat BMSCs. A,B, The expression of CircPVT1 and miR‐21‐5p were detected by RT‐PCR. C, RT‐PCR was taken to assess the levels of osteogenic differentiation markers ALP, RUNX2, CoL1a1, OCN and the expression of osteoclast‐related genes ACP5 and CTSK. D,E, BrdU and flow cytometry were conducted to examine cell proliferation and apoptosis ability. F, Western blot detection of Smad7, Smad2/3, Bax and Bcl2 protein expression. *P < .05, **P < .01, ***P < .001 (vs.con group). ##*P* < .01, ###*P* < .001 (vs.GC group). N = 3

To investigate the effect of CircPVT1 overexpression on the differentiation ability of BMSCs, we constructed an overexpression model of CircPVT1 based on GC‐treated cells and measured CircPVT1 and miR‐21‐5p expression via RT‐PCR. The results revealed that after GC treatment, CircPVT1 expression was down‐regulated, while miR‐21‐5p expression was up‐regulated (compared with the con group). On this basis, the overexpression of CircPVT1 cells were conducted and inhibited miR‐21‐5p expression (*P* < .05, Figure 3A,B). Further, RT‐PCR was conducted to monitor the expression of osteogenic differentiation markers and osteoclast‐related genes, and it was found that when the circPVT1 overexpressed, the expression of ALP, RUNX2, CoL1a1 and OCN increased, which suggests that he overexpression of CircPVT1 up‐regulated the expression of ALP, RUNX2, CoL1a1 and OCN (*P* < .05, Figure 3C), and the overexpression of CircPVT1 reduced the expression of osteoclast‐related genes ACP5 and CTSK (*P* < .05, Figure 3C). Meanwhile, BrdU and flow cytometry were implemented to examine cell proliferation and apoptosis ability, respectively. It was found that overexpression of CircPVT1 promoted cell proliferation and inhibited apoptosis (compared with the GC group) (*P* < .05, Figure 3D,E). We carried out Western blot to determine the protein levels of Smad7, Smad2/3, Bcl2 and Bax. It was found that GC markedly enhanced the activation of Smad2/3 and Bax level, while reduced Smad7 and Bcl2 level. By contrast, overexpressing CircPVT1 reduced Smad2/3 and Bax, and promoted Smad7 and Bcl2 (compared with GC group) (*P* < .05, Figure 3F). The above outcome demonstrated that the overexpression of CircPVT1 could accelerate BMSCs differentiation and proliferation and suppress their apoptosis.

### 3.4 Overexpression of miR‐21‐5p inhibited the differentiation, proliferation and promoted apoptosis of BMSCs

**FIGURE 4:**
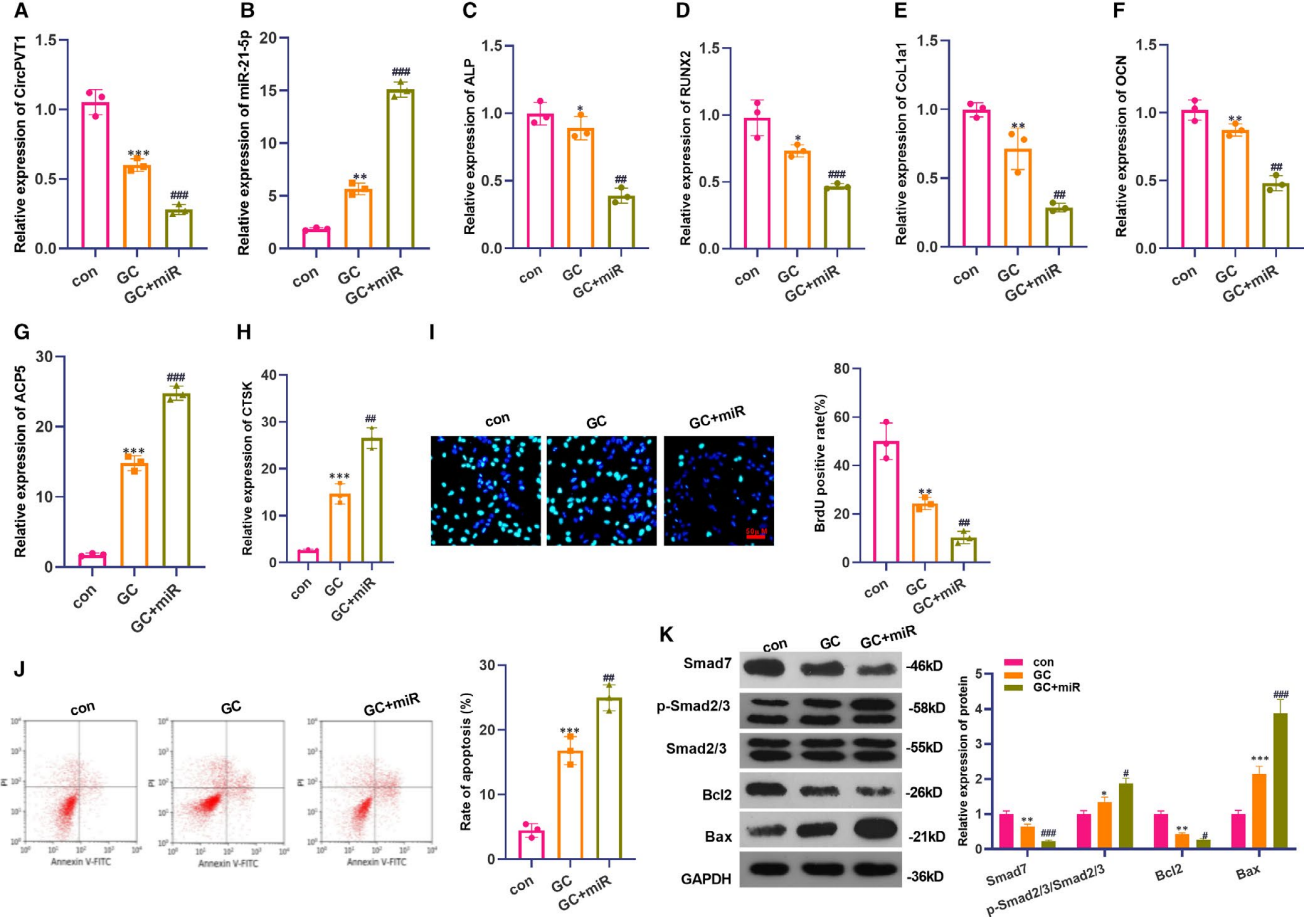
Overexpression of miR‐21‐5p inhibited the differentiation and proliferation of BMSCs and promoted apoptosis. MiR‐21‐5p mimics were transfected based on 1600 mg GC‐treated BMSCs. A,B, The expression of CircPVT1 and miR‐21‐5p were detected by RT‐PCR. C‐H, RT‐PCR was utilized to measure osteogenic differentiation markers ALP, RUNX2, CoL1a1, OCN and the expression of osteoclast‐related genes ACP5 CTSK. I‐J, BrdU and flow cytometry were taken to evaluate the effects of transfection of miR‐21‐5p mimics on the proliferation and apoptosis of BMSCs. K, Western blot detection of Smad7, Smad2/3, Bax, Bcl2 protein expression. *P < .05, **P < .01, ***P < .001 (vs.con group). ##*P* < .01, ###*P* < .001(vs.GC group). N = 3

First, on the basis of GC‐treated cells, we transfected miR‐21‐5p mimics and examined CircPVT1 and miR‐21‐5p expression via RT‐PCR. It turned out that CircPVT1 expression was down‐regulated, and miR‐21‐5p expression was up‐regulated after GC treatment (compared with the con group). On this basis, transfection of miR‐21‐5p mimics further promoted the down‐regulation of CircPVT1 and up‐regulation of miR‐21‐5p (*P* < .05, Figure 4A,B). Then, RT‐PCR was employed to examine the levels of osteogenic differentiation markers ALP, RUNX2, CoL1a1 and OCN and the expression of osteoclast‐related genes ACP5 and CTSK. It turned out that transfection of miR‐21‐5p mimics promoted the knockdown of these osteoclast‐related markers (*P* < .05, Figure 4C‐F), while the expression of osteoclast‐related genes ACP5 and CTSK was up‐regulated (*P* < .05, Figure 4G,H). Meanwhile, BrdU and flow cytometry results showed that compared with the GC group, transfection of miR‐21‐5p mimics suppressed cell proliferation and promoted apoptosis (*P* < .05, Figure 4I‐J). Western blot results manifested that after GC treatment, miR‐21‐5p mimics were transfected in BMSCs, the protein expressions of Smad7 and Bcl2 were both up‐regulated. In contrast, the Smad2/3 phosphorylation and the Bax expression were significantly impeded (*P* < .05, Figure 4K). These findings showed that transfection with miR‐21‐5p mimics inhibited the differentiation, proliferation and apoptosis of BMSCs.

### 3.5 MiR‐21‐5p was the target of circPVT1 and Smad7

**FIGURE 5:**
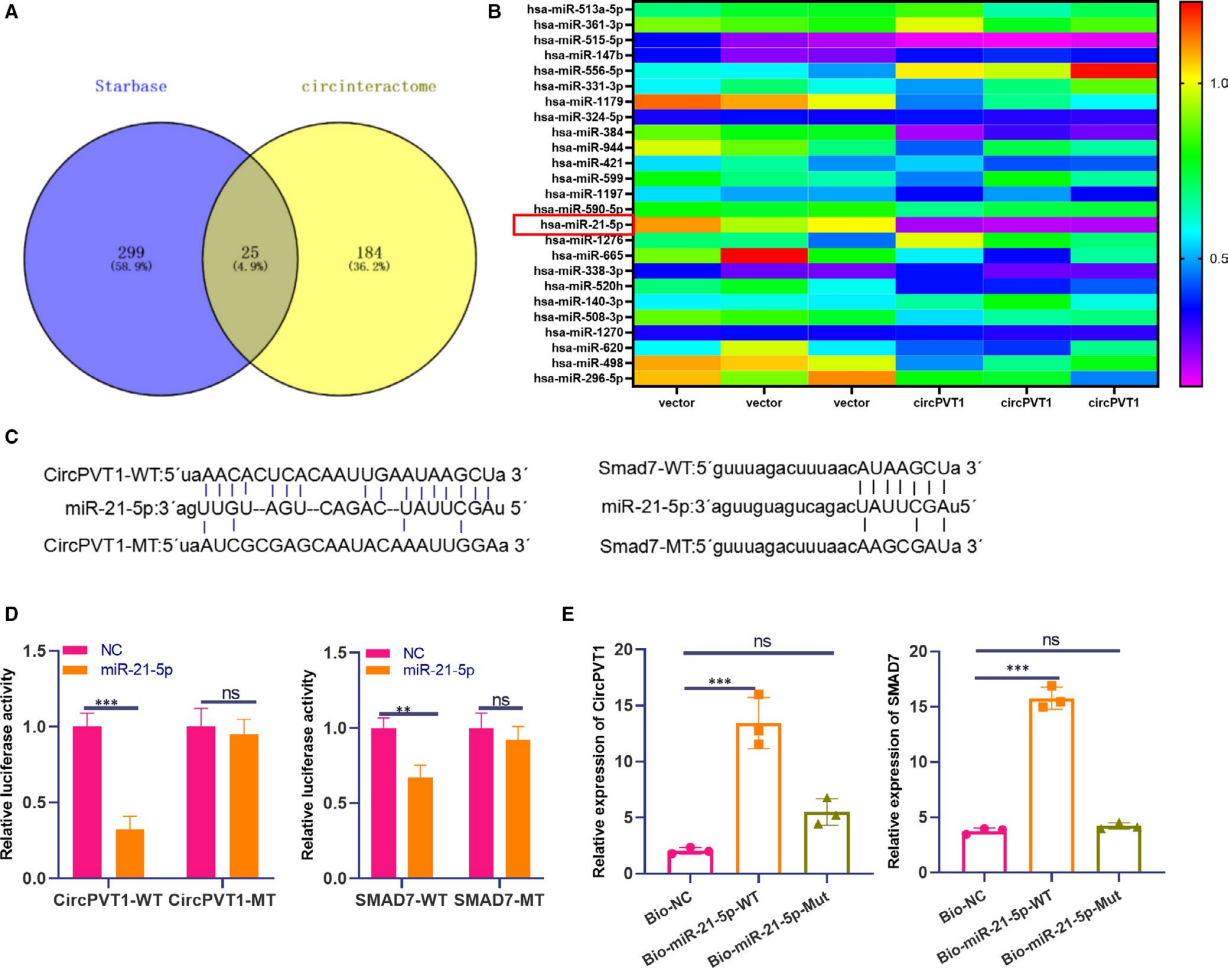
MiR‐21‐5p is the target of CircPVT1 and Smad7. A, The relevant downstream molecular targets of CircPVT1 were analysed by Venn diagram. B, The expression of miRNAs after CircPVT1 overexpression was analysed by heat map. C, StarBase database (http://starbase.sysu.edu.cn/) was browsed to predict the binding site of miR‐21‐5p to CircPVT1 and Smad7. D, Dual‐luciferase activity experiment and RNA pull‐down assay (E) confirmed the binding relationship of miR‐21‐5p with CircPVT1, miR‐21‐5p and Smad7 in BMSCs cells. **P < .01, ***P < .001, ns*P* > 0.05 (vs.NC group). ###*P* < .001, ns*P* > 0.05 (vs.NC group). N = 3

According to the lncRNA‐miRNA‐mRNA regulatory network diagram, we were urgent to understand the regulatory mechanism of CircPVT1 and Smad7. Through Venn diagram analysis, we found that 25 miRNAs were potential downstream targets of CircPVT1 (Figure 5A). Meanwhile, RT‐PCR results showed that the expression of miR‐21‐5p was suppressed most obviously after overexpressing CircPVT1 (Figure 5B). By browsing the StarBase database (http://starbase.sysu.edu.cn/), the binding sites between miR‐21‐5p and CircPVT1 and Smad7 were found and shown in Figure 5C. To clarify the targeted relationship between the three, we conducted dual‐luciferase activity experiments. And the miR‐21‐5p mimics suppressed the luciferase activity of cells transfected with CircPVT1‐WT or Smad7‐WT vectors but had no significant effect on CircPVT1‐MUT and Smad7‐MUT (*P* < .05, Figure 5D). Besides, the RNA pull‐down assay showed that CircPVT1 and Smad7 were obtained not from Bio‐miR‐21‐5p‐MUT but specifically from Bio‐miR‐21‐5p‐WT (*P* < .05, Figure 5E). This situation suggests that miR‐21‐5p contains binding sites for CircPVT1 and Smad7.

### 3.6 Up‐regulating CircPVT1 can inhibit BMSCs osteonecrosis induced by miR‐21‐5p

**FIGURE 6:**
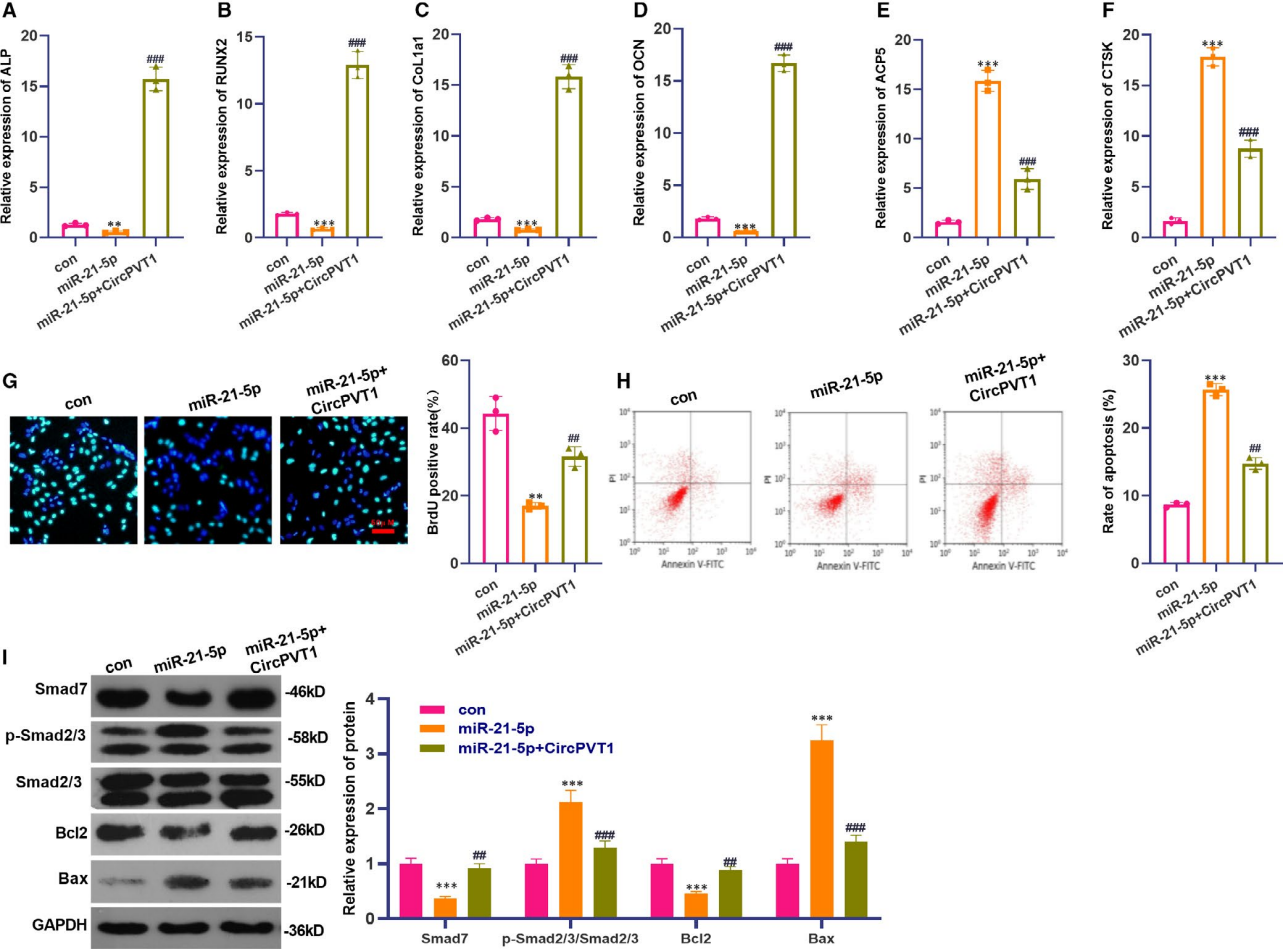
Up‐regulation of CircPVT1 inhibited miR‐21‐5p‐induced osteonecrosis of BMSCs. BMSCs were transfected with miR‐21‐5p mimics and/or CircPVT1 overexpression plasmids. A‐F, RT‐PCR was employed to assess the levels of osteogenic differentiation markers ALP, RUNX2, CoL1a1, OCN and the expression of osteoclast‐related genes ACP5 and CTSK. G‐H, BrdU and flow cytometry were used to detect BMSC proliferation and apoptosis. I, Western blot detection of Smad7, Smad2/3, Bax, Bcl2 protein expression. **P < .01, ***P < .001 (vs.con group). ##*P* < .01, ###*P* < .001 (vs.miR‐21‐5p group). N = 3

To investigate the interaction of CircPVT1/miR‐21‐5p in femoral head necrosis, BMSCs were transfected with miR‐21‐5p mimics and/or CircPVT1 overexpressed plasmids. Firstly, RT‐PCR was conducted to examine osteogenic differentiation markers ALP, RUNX2, CoL1a1 and OCN and osteoclast‐related genes ACP5 CTSK. The results illustrated that the levels of the above osteoclast differentiation markers were reduced in the miR‐21‐5p group (*P* < .05, Figure 6A‐D), and the expression of osteoclast‐related genes was up‐regulated (*P* < .05, Figure 6E,F). The levels of ALP, RUNX2, CoL1a1 and OCN in the miR‐21‐5p + CircPVT1 group were up‐regulated, while the expression of ACP5 and CTSK were decreased (*P* < .05, Figure 6A‐F). Moreover, BMSC proliferation and apoptosis were evaluated by BrdU and flow cytometry. BMSC proliferation in the miR‐21‐5p group was down‐regulated while the apoptosis was enhanced (compared with the con group) (*P* < .05, Figure 6G,H). In the miR‐21‐5p group, overexpression of CircPVT1 apparently reduced the miR‐21‐5p induced effect (*P* < .05, Figure 6G,H). Besides, after the up‐regulation of miR‐21‐5p, the protein expression of Smad7 and Bcl2 were down‐regulated, while Smad2/3 and Bax were up‐regulated. Nevertheless, the CircPVT1 overexpression exerted the opposite effect (*P* < .05, Figure 6I). The statistics manifest that miR‐21‐5p induces BMSC osteonecrosis, while CircPVT1 inhibits the miR‐21‐5p‐mediated effect.

### 3.7 The effect of overexpression of CircPVT1 on osteoblasts of femoral head necrosis in rats

**FIGURE 7:**
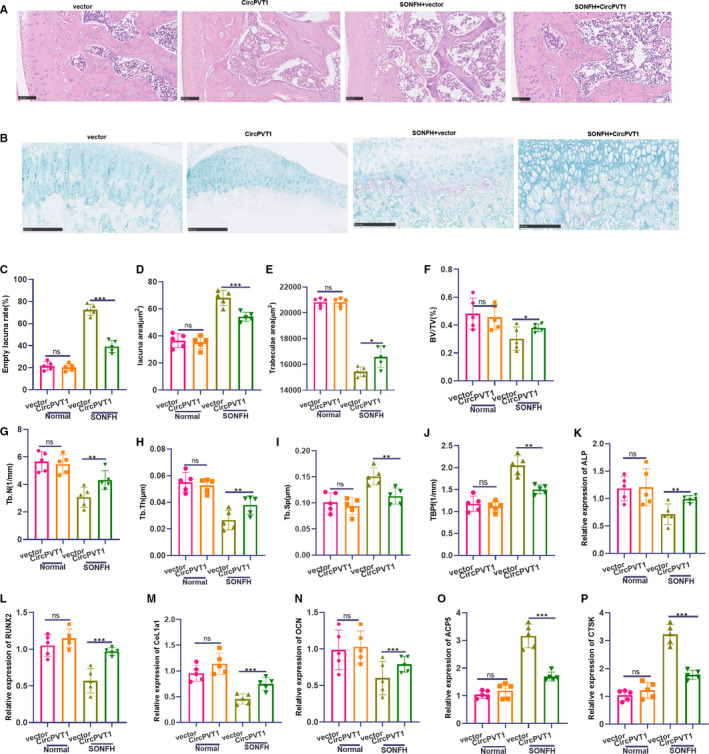
The effect of overexpressed CircPVT1 on osteogenic differentiation of rat femoral head necrosis was verified in vivo. The CircPVT1 overexpression plasmid was injected into the ventricle of SIONFH rats. A, HE staining was taken to check the pathological damage of the femur. B, TRAP staining detected osteoclast viability. C‐E, Measurement of bone cavity rate, cavity area and trabecular area. F‐J, Determination of bone volume fraction, trabecular bone quantity, trabecular bone thickness, trabecular bone separation and mode factor. K‐P, RT‐PCR was used to detect osteogenic differentiation‐related genes ALP, RUNX2, CoL1a1, OCN and osteoclastogenesis‐related genes ACP5 and CTSK. NS *P* > .05, **P < .01, ***P < .001 (vs.vector group). N = 5

To further verify the effect of CircPVT1 on osteoblastic differentiation of femoral head necrosis, we conducted experiments in vivo. The results of HE staining showed that in the SONFH model, after the overexpression of CircPVT1, the trabecular structure in the femoral head sections of the rats was slightly degenerated and set in order, bone cells filled the lacunae, bone surface folded and collapse were not noticeable, and calcification zone was well connected to the subchondral trabecular structure (Figure 7A), and TRAP activity was dampened (Figure 7B). Meanwhile, the measurement results of bone cavity rate, cavity area and trabecular area showed that the CircPVT1 group had decreased cavity rate and area, while the trabecular area increased (compared with the vector group) (*P* < .05, Figure 7C‐E). What's more, bone volume fraction, number and thickness of trabecular bone increased significantly, while trabecular separation degree and model factor decreased (*P* < .05, Figure 7F‐J). The above data of bone histomorphology manifested that the overexpression of CircPVT1 improves the bone tissue morphology. In the meantime, overexpression of CircPVT1 up‐regulated the levels of osteogenic differentiation‐related genes ALP, RUNX2, CoL1a1 and OCN (*P* < .05, Figure 7K‐N) and decreased the expressions of osteoclast‐related genes ACP5 and CTSK (*P* < .05, Figure 7O,P). The outcome demonstrated that CircPVT1 promotes BMSC differentiation in vivo and attenuates SIONFH.

### 3.8 The effect of overexpressing CircPVT1 on SMAD7 and SMAD2/3 in vivo

**FIGURE 8:**
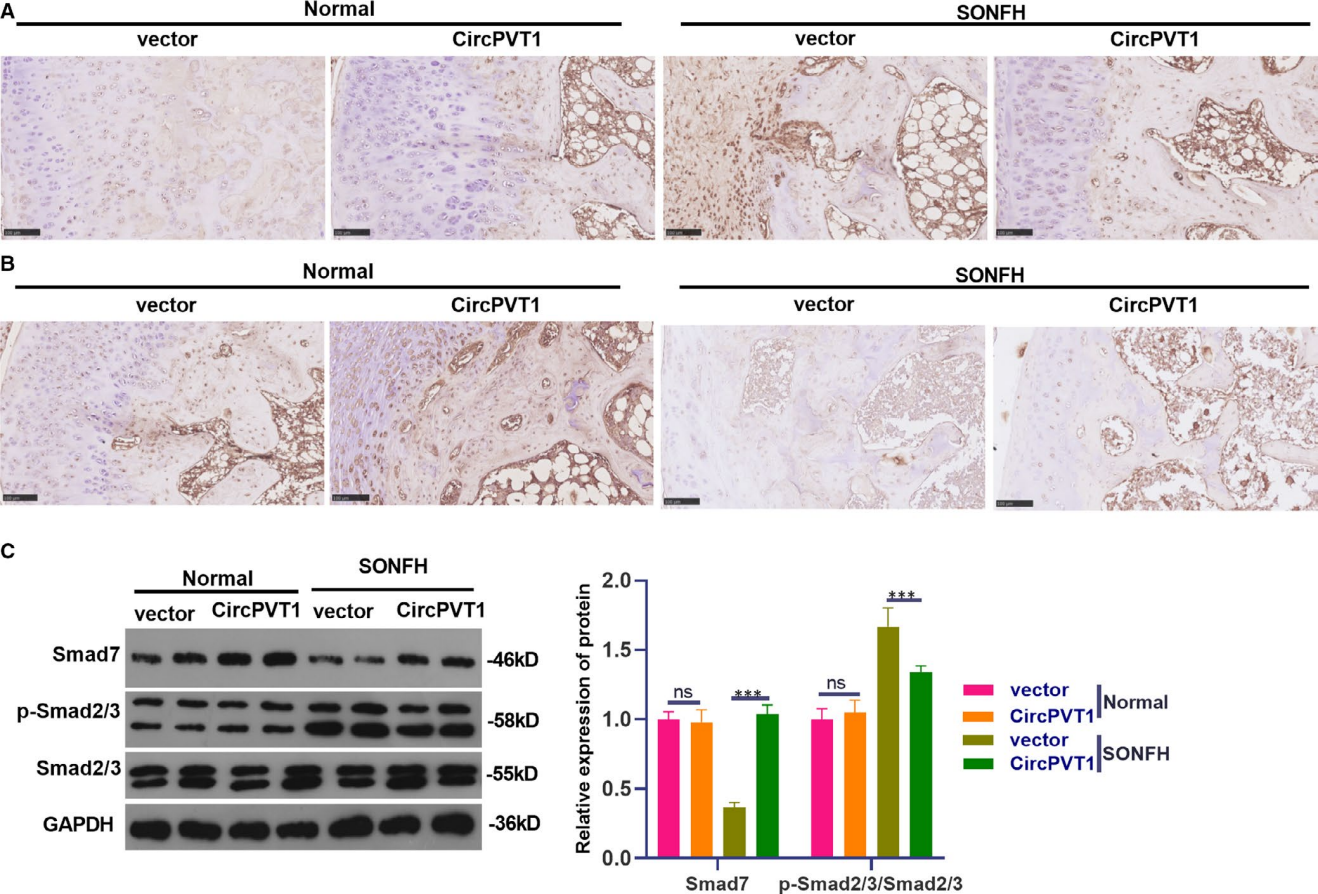
Effect of overexpressing CircPVT1 on SMAD7 and SMAD2/3 in vivo. The treatment method was the same as that in Figure 7. A,B, IHC was implemented to verify the positive expression of Smad7 and pro‐apoptotic protein Bax. C, Western blot was applied to monitor the expression of Smad7, Smad 2/3, Bcl2 and Bax. NS *P* > .05, ***P < .001 (vs.vector group). N = 5

First, we adopted IHC to detect the expression of Smad7 and the pro‐apoptotic protein Bax. We found that overexpressing CircPVT1 in the SONFH model significantly increased Smad7‐positive labelled cells (Figure 8A) and reduced Bax positive labelling cells (Figure 8B). Western blot results illustrated that there was no significant difference in Smad7 expression and Smad2/3 phosphorylation after CircPVT1 overexpression in the normal group. Nevertheless, overexpressing CircPVT1 in SONFH model significantly elevated Smad7 and Bcl2 expression and abated Smad2/3 phosphorylation and Bax level (*P* < .05, Figure 8C), indicating that CircPVT1 up‐regulated Smad7 and inactivated Smad 2/3 in vivo.

## 4 DISCUSSION

As a subtype of non‐traumatic ONFH, steroid‐induced ONFH (SIONFH) is associated with increased intra‐osseous pressure caused by bone marrow adipocyte proliferation and increased adipogenesis, which can slow the femoral head blood flow and eventually lead to ischaemic osteonecrosis.^6^, ^23^ This problem has a negative impact on the patients' life quality and prognosis. This study explored the specific molecular mechanism of CircPVT1 affecting SIONFH in a SIONFH model and found that CircPVT1 was down‐regulated in the SIONFH model, and up‐regulating CircPVT1 inhibited miR‐21‐5p expression, promoted the activation of the TGFβ/Smad7 pathway and attenuated SIONFH.

CircPVT1, as an endogenous circrRNA molecule, is located at 8q24.21. Many papers have reported that CircPVT1 makes a great different in lung adenocarcinoma,^24^glioblastoma^25^ and other tumours. However, the role of CircPVT1 in orthopaedics, especially SIONFH, has hardly been reported. The regulatory function and distinct expression profile of other CircRNA types in orthopaedic diseases are increasingly significant. Taking CircRNA DAB1 as an example, it accelerates the proliferation and osteogenic differentiation of BMSCs through NOTCH/RBPJ.^26^Wang XB et al found that Circ_0006393 promoted the osteogenesis of glucocorticoid‐induced osteoporosis by sponging miR‐145‐5p and up‐regulating FOXO1 expression.^27^ Meanwhile, Zhu et al reported that the toxicity of human osteoblasts induced by dexamethasone (DEX) was significantly correlated with the down‐regulation of CircHIPK3.^28^Additionally, CircRNA plays a significant role in rheumatoid arthritis^29^ and post‐menopausal osteoporosis.^30^More importantly, Chen G et al reported in April 2020 that there are 820 circRNAs (including 460 up‐regulated circRNAs and 360 down‐regulated circRNAs) that are differentially expressed in SIONFH‐BMSCs. Further experiments show that CircRNA CDR1, as an up‐regulated CircRNA, can exert a great effect on the inhibition of lipogenesis/osteogenesis differentiation of SIONFH‐BMSC through the CDR1AS‐miR‐7‐5P‐Wnt5B axis.^31^, ^32^In view of the above studies, we suggested that CircPVT1, as an up‐regulated or down‐regulated CircRNA, might have significant value within SIONFH through some mechanism. Fortunately, this study proved that CircPVT1 expression was down‐regulated in ONFH cells induced by GC model. Experiments in vivo and in vitro showed that overexpression of CircPVT1 reversed the femoral head necrosis of rats induced by GC, promoted BMSCs differentiation, proliferation and inhibited apoptosis, suggesting that CircPVT1, as a down‐regulated circRNA, has a significant protective action in SIONFH.

Important results in this research show that miR‐21‐5p was highly expressed in SIONFH, suggesting that miR‐21‐5p is abnormally expressed in SIONFH. The abnormal expression of miR‐21‐5p has also been found in other studies. Recent studies have demonstrated that miR‐21‐5p has significant value in osteoblast‐osteoclast coupling in vitro.^33^ For instance, Lian F et al discovered that miR‐21‐5p may be a potential bone‐promoting regulator, and icariin can prevent the Ti‐induced inhibition of osteoblasts differentiation and mineralization by up‐regulating miR‐21‐5p.^34^Meanwhile, exosome‐derived miR‐21‐5p induces abnormal bone formation in acromegaly by regulating the growth hormone/insulin‐like growth factor‐1 (GH/IGF‐1) axis.^35^However, some studies reported that the levels of miR‐148a and miR‐21‐5p in patients with type 1 diabetes are elevated and correlated with bone strength and metabolic indicators.^36^Additionally, Zhang A et al claimed that miR‐21‐5p is a key regulator of Gdf5 in chondrocytes, and knocking down miR‐21‐5p reduces cartilage matrix degradation in temporomandibular joint osteoarthritis (TMJ‐OA) by targeting Gdf5.^37^These findings reveal that the role of miR‐21‐5p in regulating osteogenic differentiation and osteoclast formation is still controversial, which is because of different regulatory mechanisms. Here, we discovered miR‐21‐5p expression was raised in SIONFH, and the overexpression of miR‐21‐5p significantly suppressed the differentiation and proliferation of BMSCS and promoted apoptosis.

More and more studies have shown that CircRNA acts as ceRNA of miRNAs and competitively inhibits miRNAs through molecular sponges, thus affecting the progression of bone diseases. For example, Shen S et al demonstrated that CircSERPINE2 binds to miR‐1271 as a competitive RNA, promotes ERG expression, reduces HCS apoptosis and promotes extracellular matrix (ECM) anabolism to prevent knee osteoarthritis (OA).^38^In addition, Chen C et al reported that CircRNA9119 alleviates osteoarthritis by regulating the miRNA‐26A/PTEN axis to inhibit the apoptosis of chondrocytes induced by IL‐1β.^39^Similar to the above studies, this study found a targeted relationship between CircPVT1 and miR‐21‐5p through database analysis, and overexpressed CircPVT1 can inhibit miR‐21‐5p‐induced osteonecrosis of BMSCs and reduce apoptosis. These findings confirmed that miR‐21‐5p, as a downstream molecule of CircPVT1, induces osteoclast formation and inhibits osteogenic differentiation in SIONFH.

TGFβ is an important cytokine involved in osteocyte function and metabolism. Previous papers have proven that TGF not only contributes to osteocyte mitosis, but also can reduce bone protein loss, increase bone deposition rate and promote osteoblast differentiation.^40^Meanwhile, Smad7, as an inhibitory Smad, is a key regulator of TGF‐β/bone morphogenetic protein (BMP) signalling through the negative feedback loop.^41^ Studies have shown that MOTS‐c promotes the synthesis of type I collagen in osteoblasts by regulating the TGF‐β/Smad signalling pathway, thus improving osteoporosis.^42^He G et al reported that miR‐877‐3p promoted osteoblast differentiation of TGF‐β1‐induced MC3T3‐E1 cells by targeting Smad7.^43^Besides, Cui H et al found that exosome‐derived miR‐21‐5p secreted by bone marrow macrophages directly targets Smad7 and causes the activation of fibrosis in tendon cells.^44^In this study, it was found that overexpressed CircPVT1 significantly suppressed the protein expression of Smad2/3 and up‐regulated Smad7, thus promoting the differentiation of osteoblasts, while overexpressed miR‐21‐5p had an opposite effect. Based on transfection of miR‐21‐5p mimics, overexpression of CircPVT1 significantly inhibited BMSCs osteonecrosis induced by miR‐21‐5p and promoted osteogenic differentiation. These results reveal that CircPVT1 can influence the development of SIONFH by regulating the miR‐21‐5p /TGFβ/Smad7 axis.

**FIGURE 9:**
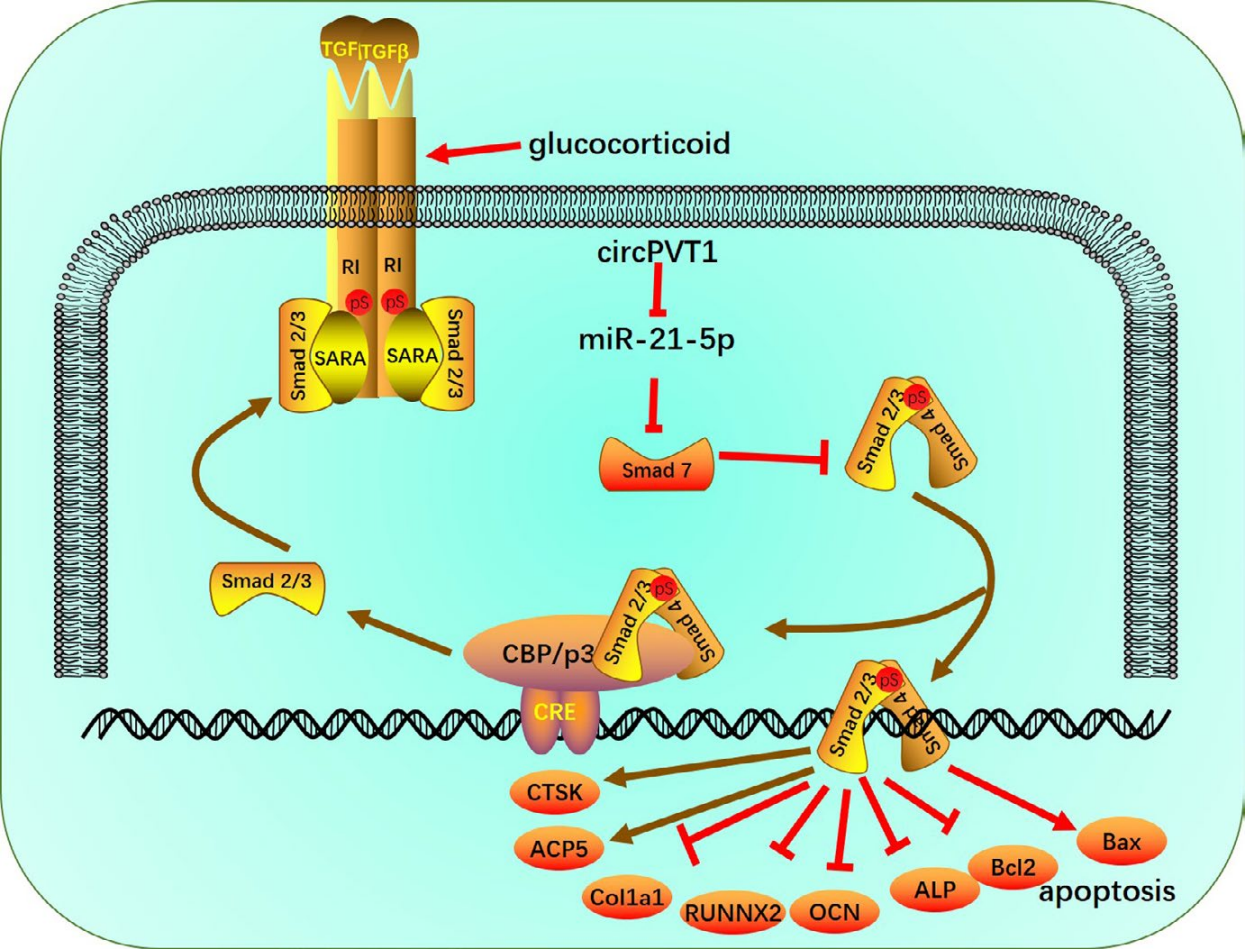
The mechanistic diagram. CircPVT1 is down‐regulated in SIONFH. Overexpressing of circPVT1 down‐regulates TGFβ/Smad2/3 expression and promotes Smad7 activation by targeting miR‐21‐5p, thereby reducing SIONFN

To sum up, our study shows that CircPVT1 is down‐regulated in SIONFH and down‐regulates TGFβ/Smad2/3 expression and promotes Smad7 activation by targeting miR‐21‐5p, thereby reducing SIONFN (Figure 9). This study provides a new reference for the diagnosis and treatment of SIONFH, but more relevant mechanisms of action and treatment strategies need to be further explored.

## CONFLICT OF INTERESTS

The authors declare that they have no competing interests.

## AUTHOR CONTRIBUTION

**Yangquan Hao:** Funding acquisition (lead); Writing‐original draft (lead). **Chao Lu:** Data curation (equal); Writing‐original draft (equal). **Baogang Zhang:** Methodology (equal); Writing‐original draft (equal). **Zhaochen Xu:** Formal analysis (equal); Investigation (equal). **Hao Guo:** Visualization (equal). **Gaokui Zhang:** Project administration (equal).

## ETHICAL STATEMENT

Our study was approved by the Ethics Review Board of Honghui Hospital, Xi'an Jiaotong University Health Science Center.

## Data Availability

The data sets used and analysed during the current study are available from the corresponding author on reasonable request.
